# Social accountability and health systems’ change, beyond the shock of Covid-19: drawing on histories of technical and activist approaches to rethink a shared code of practice

**DOI:** 10.1186/s12939-022-01645-0

**Published:** 2022-03-25

**Authors:** Erica Nelson, Peter Waiswa, Vera Schattan Coelho, Eric Sarriot

**Affiliations:** 1 The Institute of Development Studies, Health and Nutrition Cluster, Brighton, UK; 2 Makerere University School of Public Health, Kampala, Uganda; 3 Centro Brasilero de Análise e Planejamiento, São Paulo, Brazil; 4 Gavi, the Vaccine Alliance, Geneva, Switzerland

**Keywords:** Social accountability, History, Health systems, Multidisciplinary Approaches

## Abstract

**Background:**

Recognition of the value of “social accountability” to improve health systems performance and to address health inequities, has increased over the last decades, with different schools of thought engaging in robust dialogue. This article explores the tensions between health policy and systems research and practice on the one hand, and health equity-focussed activism on the other, as distinct yet interacting processes that have both been impacted by the shock effects of the Covid-19 pandemic. This extended commentary brings multidisciplinary voices seeking to look back at health systems history and fundamental social-institutional systems’ behaviors in order to contextualize these current debates over how best to push social accountability efforts forward.

**Analysis:**

There is a documented history of tension between long and short processes of international health cooperation and intervention. Social accountability approaches, as a more recent strategy to improve health systems performance, intersect with this overarching history of negotiation between differently situated actors both global and local on whether to pursue sustained, slow, often community-driven change or to focus on rapid, measurable, often top-down interventions. Covid-19, as a global public health emergency, resulted in calls for urgent action which have unsurprisingly displaced some of the energy and aspiration for systemic transformation processes. A combination of accountability approaches and mechanisms have their own legitimacy in fostering health systems change, demanding collaboration between those that move both fast and slow, top-down and bottom-up.

**Conclusion:**

We argue that social accountability, much like all efforts to strengthen health systems, is “everybody’s business” and that we must understand better the historical processes that have shaped the field of practice over time to move forward. These differences of perspective, knowledge-base and positioning vis-a-vis interventions or longer-term political commitment should not drive a conflict of legitimacy but instead be named, subsequently enabling the development of a shared code of conduct that applies to the breadth of actors involved in social accountability work. If we are concerned about the state of/status of social accountability within the context of “building back better” we must approach collaboration with a willingness to create dialogue across distinct disciplinary, technical and politically-informed ways of working.

## Background

Over the last two decades, there has been increasing use of “social accountability” language and practices in global health, particularly in the area of health policy and systems research and interventions. In our use of the term “social accountability” in a contemporary context we refer to the broad range of strategies that are used to bolster community engagement and to hold public and private actors to account within the context of the public health sector, while noting that many health systems are pluralistic in nature and not wholly (or even partially) publicly controlled or owned. Social accountability organizes constituencies and information to generate dialogue or confrontation, and can aim to do different things, such as: address equity issues, quality of care, and unequal power relationships between citizens, care providers, health systems managers, and policy makers. Alternatively it can be mobilized to draw attention to service providers’ blind spots and/or broken public commitments. This extended commentary explores the tensions, actual or perceived, between health policy and systems research interventions and program implementation on the one hand (the world of “technicians”), and health equity-focussed activism on the other (the world of “activists”), as distinct yet interacting fields that have both been impacted by the shock effects of the Covid-19 pandemic. We use “technicians” and “activists” as short-hand for schools of thoughts or patterns of approach, recognizing that these terms are an over-simplification of what is in fact a more complex reality of shifting roles and strategies.

With increased global health attention to social accountability, health program actors have moved closer to the spaces where health equity activists, local, ‘global’ and in-between, articulate their demands for accountability on governments and public duty-bearers. The authors of this commentary are part of the Community of Practice (CoP) on measuring social accountability and health outcomes convened by the Department of Sexual and Reproductive Health and Research based at the World Health Organization. As researchers coming to this topic with different foundational framings – a social historian and participatory ethnographer (EN), a health systems researcher working within a national context where the history of health systems creation marked a political turning point (VS), and health systems researchers and practitioners both internationally and nationally situated (ES, PW)— we have seen these two fields of activity - health systems research and practice, and health equity-focussed activism—develop over time as distinct, yet interacting, processes. More recently, we have witnessed the Covid-19 pandemic as a ‘shock event’ that has disrupted the world and subsequently these fields of activity in which we, the authors, are differently involved. The wide range of public health and health systems responses to Covid-19 have taken center stage, resulting initially in a the dominance of command-and-control responses, while participatory and community-led responses emerged more slowly, or rather, with less international attention [1]. Within this context, social accountability for health equity efforts are in a state of flux. This paper constitutes a dialogue between our distinct disciplinary backgrounds, institutional positions, fields of practice and orientation to social accountability debates, with the aim of calling attention to the patterns of thought and action that have shaped what now constitutes social accountability work in global health. We start by taking a historical perspective showing that questions about social accountability fit within a larger (and longer) story of competing approaches to international public health and unresolved tensions between them. We then outline our understanding of social accountability as a core feature of social and institutional system. We explore the nature of the difference between at the “technicians” and “activist” schools of thought and practice in current debates, and conclude with a set of principles that may better guide critical reflection on the legitimacy, limitations, and risks inherent to different approaches and the imperative to find common ground in light of the health, economic, societal and political impacts of Covid-19.

Given the already-existing challenges in maintaining focus and momentum across a diverse range of social accountability efforts in global health, we seek to look back, look forward, and discern what this moment offers in terms of strengthening an equity-focussed agenda.

## Social accountability, the long and short of it

Historical perspectives can help us better understand the current moment for social accountability in health. Accountability theorists and health systems researchers talk about the importance of historical context, but there is often little effort made to deepen understandings of how the past has shaped the systems, structures and logics of the present. International development and global health actors, naturally engaged in planning and forecasting, can have tremendous blindspots when it comes to the past failures and unmet promises of their own institutions or within their fields of practice [2–5]. One outcome of the ahistoricity of these fields is that shared understandings or consensus narratives of the recent past—whether that be a political cycle or just the last timebound project—are decoupled from the more distant past, which leaves unexamined the potential threads of connectivity between them [6].

This is not the place to elaborate on the breadth of historical research that could reasonably inform current social accountability approaches, but rather a reflection on the value of integrating long- and short-term perspectives in social accountability processes, as well as giving attention to intergenerational commitments to social justice. Short-term versus long-term ways of working have emerged in different areas of global health: service delivery and capacity building, system strengthening, technically-driven health interventions and community-engaged, participatory public health approaches. It is important to recognize that the tensions between these distinct modes of practice are not new phenomena in international health cooperation. Though this commentary is not a bibliographic review, it draws on one authors’ (EN’s) historical research over time related to international public health/global health, as well as an intergration of historical perspectives into a contemporary health research practice. This commentary reflects an emerging and expanding field of literature that has identified some of the origins of, and the repeating nature of, tensions between “activist” and “technical” approaches to health from the interwar period through to the turn of the twenty-first century [2–9].

We begin with a brief review of relevant historiography on the emergence of “health systems” and “accountability” as concepts and as fields of practice. Prior to the spread of “health systems” approaches in international public health (circa mid 1960s), transnational networks of public health/sanitarian activists and technicians recognized the interrelationship between local infrastructure, sanitation, education, economic status, agricultural development, nutrition as linked to health outcomes. As early as 1937, at the Bandung “Intergovernmental Conference of Far Eastern Countries on Rural Hygiene” one can identify the emergence of “intersectoral and interagency cooperation” across fields of practice, distinct disciplines, and areas of expertise with the aim of addressing shared public health challenges [10]. The identification of tensions between “vertical” (disease-oriented, top-down) and “horizontal” (community-led, multidisciplinary) approaches to public health, and articulation of a “system” of health in which all approaches could be conceivably encompassed, was first articulated by Carlos González of Venezuela’s Ministry of Health and Social Welfare in a 1965 background paper written on behalf of the World Health Organization’s (WHO) “Study Group on the Integration of Mass Campaigns Against Specific Diseases into General Health Services” [9, 11].

In 1967, the WHO set up the in-house Division of Research in Epidemiology and Communication Sciences (RECS) to develop new planning methods for health “systems” in “developing” countries, led by Kenneth Newell, and drawing on the experience and expertise of innovators in multidisciplinary planning approaches from diverse country contexts [9]. In 1975, Newell launched *Health by the People,* an edited compendium of community public health initiatives which highlighted the grassroots work of social medicine activists in Latin America, South East Asia, the Middle East and China [12]. For the sake of brevity, this is a top-line reading of what was in fact a dynamic transnational movement of interconnected health systems thinkers, practitioners and community public health activists. However, we outline these few key moments in the chronology to give some sense of the expanse of time in which community-based - or what might now be called “people-centred” approaches - to health systems development and change were being first developed. Thus, in 1978 when the Declaration of Alma Ata called for community-led approaches to health systems development, in addition to rearticulating health as a human right, this marked the culmination of fifty-plus years of international health cooperation and learning [13].

The Alma Ata moment, contrary to how it is portrayed in global health literature, marked the beginning of a shift away from “horizontal” or community-led, multi-sectoral, ‘activist- inclusive approaches to health systems development within international/global health circles. By 1979, within the WHO, UNICEF and amongst Western global health leadership, the tide had turned towards a politically neutral and economically feasible “selective” approach to Primary Health Care [3, 8, 14, 15]. With a global economic downturn, increased restrictions on development aid, the rise of neoliberalism and conocomitantly new logics of efficiency and efficacy at scale, the period of the mid 1980s to late 1990s saw those who advocated sustained, grassroots processes of change in public health lose ground to those focused on disease or health-issue specific interventions and increasingly siloed areas of expertise [8, 16]. Within this overarching context, the World Bank, which by the mid-1990s was a leading actor in global health and development, began to experiment with “pro-participation” approaches to public service planning processes, incorporating the language of Alma Ata, while at the same time promoting metrics-driven, top-down (“vertical”) health interventions [17]. With the 2004 publication of the World Development Report, pro-participation approaches, renamed “social accountability” went from being a practice emergent in public health activist circles to a set of techniques aimed at addressing short-term health systems change [18].

The development of systems thinking and the tensions between “activist” and “technician” led approaches to public health are important precedents to understand contemporary accountability ecosystems framings. Over time, conflicts have emerged between health systems strengthening researchers and practitioners on the one hand, and health equity-focussed activists on the other, the former associated with timebound project cycles and the latter with generational societal transformations [19, 20]. A “whole systems approach” takes into account a broader set of influencing actors, institutions and their interrelationships on health outcomes, as well as the mechanisms that connect grassroots or community-led efforts to changes at higher levels of public health decision-making and resource allocation [18]. Within this “whole system” approach the value of differently positioned, and differently skilled actors pursuing accountability for health equity is clear. Thus, we don’t argue here in favor of either “technician” or “activist” approaches to social accountability, but instead unpack the tensions between them and why they might exist, and how to draw on both/all ways of working within different time-scales of change.

Prior to the the Covid-19 pandemic, accountability theorists and practitioners in health systems were concerned with the rise of digital technologies, the increased corporatization of health systems and the concomitant challenges of embedding accountability mechanisms within institutions, regulatory powers, professional associations, and at an international level – a shifting group of global health governance leaders. Within the context of the Covid-19 crisis, as with most emergencies, the first instinct has been to focus on short-term response and “command and control” interventions, at the expense of measures which may have provided more community engagement and involvement in the development of prevention and risk management strategies [21]. While there is no question that we have needed the rapid scaling of personal protection, social distancing, progress in management of respiratory distress in intensive care, and rapidly developed and scaled vaccine strategies, we are also well aware of the need for adequate resourcing of health systems broadly speaking, and attention to ways in which health systems continue to underperform for the most marginalized and vulnerable populations [21, 22].

Without knowing the future development of the Covid-19 pandemic, we know that it will not be the last pandemic or global health emergency [23]. To date, it has triggered a number of old dysfunctions in ways that are potentially damaging to social accountability efforts—for example when the space for critical engagement between health duty-bearers and service recipients has shrunk and when communities have been left out of critical decision-making processes. At the same time, it has made clear the necessity of social accountability oriented towards the achievement of greater health equity. When the longer historical record of international public health cooperation is taken in account, it is easier to place social accountability efforts – both technician and activist-led – within a context of unresolved tensions between distinct ways of thinking about and addressing health systems challenges. These debates are not merely theoretical or intellectual in nature, but are reflected in contemporary policy processes and development assistance for health financing.

## Accountability meanings and practices in contemporary health systems work

Accountability takes many forms (administrative, managerial, legal-political, market-driven) of which social accountability is just one element [18, 24]. In the health sector, activists and technicians tend to focus their efforts where managerial, administrative, legal-political forms of accountability are failing. Social accountability approaches commonly aim to give “teeth” to other forms of accountability [25–27]. Activist approaches look to social accountability as a means to rebalance or shift power dynamics, but in the specifics, they often relinquish the responsibility to managerial and administrative forms of accountability, mobilizing again when new gaps or shortcomings appear. Health systems technicians, especially those operating at the transnational and international level of cooperation, commonly intervene through time-bound projects and investments, attached to measurable outcomes. They do not seek to replace existing forms of administrative accountability, but often try to spur improvements in efficacy and impact. While there can be stark differences in the positioning, perspective and methods of these distinct categories of actors, what we label here as “technicians” and “activists”, they share the belief that accountability is a non-negotiable element of a functioning social and institutional (in this case, health-focussed) system [27–30]. For illustrative purposes, Tables 1 and 2 provide a short summary of the experience of VS and PW, respectively in Brazil and Uganda.

**Table 1:** Social-change (“activist”) approach to social accountability: an example from Brazil

The overarching history of the Brazilian experience of social participation in health systems development and reforms exemplifies a long-term approach to social accountability. In the 1970’s a coalition of popular movements, national and international organizations launched and supported a national health movement. In the context of a post-military dictatorshiop redemocratization process, which stretched between the 1980s and 1990s civil society groups fought successfully for the institutionalization of social participation as part of the Brazilian universal health care system (*Sistema Único de Saúde,* otherwise known as the SUS). From the 1990s to today, this social participation process has resulted in the creation of more than 5000 health councils with nearly 100,000 individual participants, in additiona to related associations. These councils serve as fora in which citizens together with service providers and public officials work together to define public policies and oversee implementation processes [31]. The councils’ planning and monitoring activities inform and are informed by social accountability efforts, in addition to the work of these councils defending access to health care on constitutional grounds. The articulation of a universal ‘right to health’ was a key component in the historical creation and implementation of the SUS, but it has also been central to the increased resourcing of, and expansion of, public health services over the last two decades. The “activist” approach to social accountability of health systems has in this way contributed to the widening of the SUS and concomitantly a reducation of health inequalities on a national scale [32, 33].

**Table 2:** Project (“technician”) approach to social accountability: an example from Uganda

In Uganda, the Community and District Empowerment for Scale-up (CODES) project launched in 2015 for a 3 year period with the aim of strengthening the management of district-level health systems to improve child survival. CODES was implemented through health districts in partnership with UNICEF and Makarere University, and community-based organizations (CBOs) engaged in health services monitoring and social accountability processes (one of three pillars of the intervention strategy). Health districts were encouraged to solicit feedback from communities related to issues of health services quality and coverage as they related to an identified set of priority health issues. This feedback was organized by CBOs through Community Dialogues, Citizens Report Cards, and SMS surveys of community member perspectives [34, 35]. A cluster randomized trial design was able to show improvements over time in the treatment of malaria, pneumonia, diarrhea, and stool disposal. Though the results of the intervention were positive, the short-term nature of the study left often questions of sustainability and potential institutionalization of social accountability mechanisms. One key learning that emerged was the important role of traditional and social media in shifting institutional norms related to the value of collaborative and community-engaged accountability. However, long-term impact may demand different intervention and institutionalization timeframes particularly where community engagement in social accountability processes and mechanisms for political accountability are less developed.	

We can now examine where perspectives diverge, in spite of this common ground. Social accountability activists, whether local, national or transnational in practice, see problematic hierarchies of power, broken institutional commitments, or at a minimum public service provider blind spots, as a starting point for change. They may focus on health systems changes specifically, but will often look more broadly at societal arrangements and structural inequalities to determine which issues matter most, to whom, and how they should be addressed. By contrast, heath systems technicians, be they working for on behalf of donor governments, philanthropic funders, or working as project implementers, may perceive gaps in performance relative to the investments made by the institutitions or governments for whom they work and be expected to address and resolve problems that have proved intractable to prior health systems professionals. Within the field of what is still considered “global” health, funding for health systems strengthening (through social accountability or other means) has long been, and remains a small slice of the overall funding pie, though by comparison with the resources available to local civil society organizations and activist groups, these funds are relatively substantial [36].

Table 3 summarizes the distinct perspectives between the two sets of actors, bringing to the surface what might otherwise remain implicit differences of approach with the aim of helping social accountability advocates of all types to find productive common ground and a forward path.

**Table 3:** Differences between two concepts of social accountability (drawing on [36–39])

	Social accountability “activist” approaches	Social accountability “technician” approaches
**Relation to government**	Direct demands, politically and power-aware, informed by local contexts, often community-led and responsive to change	Negotiated demands, likely consensus oriented or veering towards politically “neutral”, more tightly tied to project objectives and accountability to funders
**Expected results**	Sustained engagement to achieve change with moments that are “seized” when advantageous	Short time frames with measurable outcomes, but aspiring to contribute to sustainability/institutionalization
**Relation to people**	Organizing citizens and community members (including non-citizens) to hold duty bearers to account	Organizing health service *beneficiaries* or “users” to become more active participants in accountability processes
**Financial resources**	Fragmented and/or inconsistent levels of funding to support activities, both endogenous and exogenous financial resources	Financing attached to project life cycle or a contained program of work; predominantly exogenous financial resources
**Technicity (definition of services and metrics)**	Diverse forms of expertise and knowledge, including experiential and indigenous, not necessarily recognized or valued within global health	Professionalized, in certain instances regulated (e.g. medical training), forms of expertise that are rewarded and valued within global health
**Power Awareness**	Explicit concern	Often secondary to demonstrating effectiveness or impact
**Accompanying measures (service standards, system support, capacity building, etc.)**	Can rely on system’s own resources and organization, though cross-border/international cooperation and learning a common element	Supportive external investment

Most dichotomies oversimplify and Table 3 is no exception. That said, what this table captures are the ways that each set of approaches have the potential to pull towards opposing extremes, when what is needed is more work that builds bridges between health systems duty bearers, funding bodies and institutions, and health equity activists [37–39]. Historically speaking, the confluence or divergence of controlled or organic, fast or slow drivers of change in health systems has been contextually-specific and dynamic (meaning, unpredictable). What has remained true over time, to return to the historiographic review with which we began, is the challenging nature of working with an awareness of both “fast” and “slow” processes of change, historically-rooted yet contemporarily experienced dynamics of power, both locally specific and internationally governed accountability initiatives when seeking health systems change.

## Discussion: a pressing need to move forward, again

The Covid-19 pandemic triggered a ‘crisis’ response as global health emergencies often do [1, 21, 23, 40]. Within the context of international public health cooperation and national-level public health response, those that have been working towards greater social accountability for health equity have experienced Covid-19 from diverse positions, some as front-line health care providers, some as activists and organizers of mutual aid responses, some supporting health systems responses more or less remotely, in greater or lesser personal safety and teleworking opportunities.

Those concerned about the state of social accountability efforts within the context of pandemic response and beyond must learn from our international public health forebearers the value of both ‘technicians’ and ‘activists’ in driving health systems change. In a context of rapid change, including processes already underway prior to Covid-19 such as shifts in global political leadership and the emergence of increasingly mixed health systems, accountability efforts aimed at addressing health inequities are all the more crucial.

In considering the impact of Covid-19 within the much longer time scale of international public health cooperation and health systems development, we suggest that social accountability should be pursued *prior* to, or *in anticipation* of, future disruptions and shocks. Those working in the social accountability space may have different methodological tools and experiential knowledge to contribute, but without drawing on the fullest spectrum of human creativity and problem-solving capacity, the effort to address longstanding health systems inadequacies will remain stunted.

## Conclusion: toward a code of practice?

Staying with the construct social accountability “technicians” and “activists” can we discern a shared set of values or “code of practice” to help shift towards more effective collaboration? We conclude with some suggestions.

“First do no harm” and “building synergies” remain valid principles, although their operationalization in practice is debated. A step in this direction may be to accept the plurality of our perspectives, and recognize the legitimacy of differently-framed agendas and diverse knowledges. Working across distinct academic disciplines, diverse forms of technical and professional expertise, and institutional cultures, let alone meaningfully incorporating indigenous and non-Western knowledge and practice, requires humility, openness and a willingness to be made uncomfortable when confronted with the limitations of one’s own assumptions. That said, when we cast our eye over the broad sweep of health systems history and the longstanding tensions between those who proffer “vertical” (top-down, disease- or health-issue oriented) versus “horizontal” (systems-wide, socially-oriented, community-engaged) approaches to public health, it is clear that there is no one model way of working. Social accountability efforts oriented towards improving health equity demand dialogic and relational approaches from both “technicians” and “activists” even if at times this dialogue proves oppositional and fraught [41].

For activists, the attention of “technicians” is an acknowledgement of their own success in demonstrating the value of social accountability to improving meaningful community engagement in health systems improvements. In seeking alliances to achieve time-bound targets with the support and funding of external interventions, social accountability activists can still leverage these opportunities for learning and coalition building, while recognizing that cooptation—institutional or personal--are ever-present challenges. On the other hand, donor agencies, health systems planners and managers of externally-funded health projects intervening in social accountability processes should recognize the disruption that their short-term work brings. Such technicians should be more attentive to the history of past health systems efforts both specific to the contexts where they work, as well as within the broader landscape of international public health cooperation before steaming ahead with “new” innovations in design and approach. Respect for endogenous processes and local perspectives should be an active and ongoing commitment. What this history teaches us is that working towards a collaborative “middle” ground is an ongoing learning process, one that is not devoid of confrontation or dissent. As with all health systems strengthening interventions, further illuminated by the wide-ranging impacts of Covid-19, social accountability is “everybody’s business” [42]. To do better than our forebearers in international public health cooperation, it is necessary to maintain awareness of how the past has shaped the present, how what what appears ‘short term’ might in fact just be an inadvertent repetition of past patterns, and that meaningful change requires ongoing commitment to open- if challenging – dialogue between distinctly positioned actors.

## Data Availability

This commentary piece is based wholly on publicly available secondary materials (e.g. journal articles, reports and commentaries in the public domain). There were no original data or supplementary materials generated or consulted in the creation of this commentary.

## Abbreviations

CBOs: Community Based Organizations
CODES: Community and District Empowerment for Scale-up
CoP: Community of Practice
RECS: Division of Research in Epidemiology and Communication Sciences
SMS: Short Message Service
SUS: *Sistema Único de Saúde* or Universal Health System
UNDP: United Nations Development Program
UNICEF: United Nations International Children’s Emergency Fund
WHO: World Health Organization
